# Carbonic Anhydrase IX Suppression Shifts Partial Response to Checkpoint Inhibitors into Complete Tumor Eradication: Model-Based Investigation

**DOI:** 10.3390/ijms241210068

**Published:** 2023-06-13

**Authors:** Julia Grajek, Jan Poleszczuk

**Affiliations:** Nalecz Institute of Biocybernetics and Biomedical Engineering Polish Academy of Sciences, 02-109 Warsaw, Poland; jgrajek@ibib.waw.pl

**Keywords:** CAIX, immunotherapy, immune checkpoint inhibitors, mathematical model, combination therapy, acidosis, resistance

## Abstract

Immune checkpoint inhibitors (ICIs) have revolutionized the treatment of solid malignancies, including non-small-cell lung cancer. However, immunotherapy resistance constitutes a significant challenge. To investigate carbonic anhydrase IX (CAIX) as a driver of resistance, we built a differential equation model of tumor–immune interactions. The model considers treatment with the small molecule CAIX inhibitor SLC-0111 in combination with ICIs. Numerical simulations showed that, given an efficient immune response, CAIX KO tumors tended toward tumor elimination in contrast to their CAIX-expressing counterparts, which stabilized close to the positive equilibrium. Importantly, we demonstrated that short-term combination therapy with a CAIX inhibitor and immunotherapy could shift the asymptotic behavior of the original model from stable disease to tumor eradication. Finally, we calibrated the model with data from murine experiments on CAIX suppression and combination therapy with anti-PD-1 and anti-CTLA-4. Concluding, we have developed a model that reproduces experimental findings and enables the investigation of combination therapies. Our model suggests that transient CAIX inhibition may induce tumor regression, given a sufficient immune infiltrate in the tumor, which can be boosted with ICIs.

## 1. Introduction

The advent of immune checkpoint inhibitors (ICIs) was a pivotal turning point in the history of cancer treatment. Unprecedentedly, monoclonal antibodies blocking the immune checkpoints PD-1 and CTLA-4 even prolong the survival of patients with advanced, metastatic malignancies [1,2]. Nevertheless, intrinsic or acquired resistance to these drugs prevents some patients from benefiting from this therapy [3,4]. Identifying the drivers of this resistance and potential combination therapy targets would allow us to fully harness the power of ICI treatment by turning non-responders into responders.

In [3,5], the predictive biomarkers of ICI therapy have been comprehensively reviewed, including PD-L1 expression on cancer cells and other cells in the tumor microenvironment (TME), tumor mutational burden and other genetic and epigenetic factors, microbiome composition, IFN-γ signatures, and the composition and distribution of tumor-infiltrating lymphocytes. Ultimately, the authors concluded that no definite marker distinguishing responders from non-responders has been identified yet. Furthermore, it is believed that only a panel of markers would have sufficient predictive power, and the search for potential drivers of resistance is still ongoing.

In non-small-cell lung cancer, CTLA-4- and PD-1-blocking antibodies have shown promising anti-tumoral activity, either alone or in combination with chemotherapy [1,6]. Importantly, ICIs induced significant and durable responses in a subset of patients with chemotherapy-refractory disease [6]. Unfortunately, the response rate across all trials was relatively low at around 20% [7]. Among PD-L1+ patients or patients treated with a combination therapy of anti-PD-1 and anti-CTLA-4, the response rate was higher, but still more than half of the patients were resistant to the therapy [7], indicating a need for further investigation of resistance mechanisms.

An emerging factor impacting treatment resistance is TME acidosis, which suppresses the immune response and selects for more aggressive, treatment-resistant cancer cells, thereby helping cancer escape immune surveillance [8]. Tumors generally have an acidic extracellular space, with pH values in the TME amounting to around 6.5–7 [8], partially due to tumor hypoxia and the cancer cells’ aberrant metabolism. Additionally, cancer cells express several molecules, such as carbonic anhydrase IX (CAIX), which assist in the acidification of the TME [9]. These enzymes catalyze the reversible hydration of $CO_{2}$, leading to an accumulation of protons [10]. Importantly, they are an attractive treatment target since they are overexpressed in many cancers, e.g., they are highly expressed in more than one-third of non-small-cell lung cancers (NSCLCs) [11]. Meanwhile, they are rare in healthy tissue [12]. Hence, inhibiting them might reinvigorate the immune response in the TME without causing unwanted side effects in the rest of the body. Currently, a small-molecule inhibitor of CAIX called SLC-0111 is undergoing clinical trials [13]. Studies investigating the combination of ICIs and CAIX inhibition, however, are still pre-clinical. In [14,15], the effectiveness of anti-CAIX CAR T-cells secreting anti-PD-L1 was shown to decrease tumor size in mice models and prevent metastasis in clear-cell renal cell carcinoma. In [16], Chafe et al. showed decreased tumor growth in mouse tumor models when combining it with anti-PD-1 and anti-CTLA-4, unlike anti-CAIX monotherapy or ICI therapy alone. In [17], we used a computational model to investigate the impact of CAIX expression on the TME and the effectiveness of anti-PD-1 with anti-CAIX. Our model simulations suggested that patients with CAIX-expressing tumors would benefit from dual inhibition with anti-PD-1 and anti-CAIX, regardless of pre-treatment PD-L1 expression, making it an independent marker.

In this study, we set out to expand on our previous work. Here, we have developed a differential equation model informed by pre-clinical data. Our main goal was to not only analyze the effectiveness of combination therapy with anti-CAIX, anti-PD-1, and anti-CTLA-4, as in the previously mentioned studies, but also the durability of the response after stopping the treatment. Furthermore, given a few realistic assumptions, we were able to analyze the model’s asymptotic behavior mathematically, which was impossible for the complex hybrid model presented in our previous study [17]. Finally, we wanted to show that our model could quantitatively reproduce experimental findings. Thanks to its computational efficiency and interpretable parameters, the proposed model can be easily calibrated to represent different solid tumor types and analyze ICI and anti-CAIX effectiveness.

## 2. Results

### 2.1. Phase Portrait Analysis Reveals Two Possible Steady States

In order to gain a deeper understanding of the qualitative behavior of our model, we analytically analyzed the number of steady states of the simplified model (16) and deduced the following proposition:

**Proposition 1.**  *The point (0,0) is a steady state of the system* (16). Depending on parameter values, the system might also have a positive steady state ($S^{*}$, $ES^{*}$).

**Proof** **of Proposition 1.** First, let us assume that S>0, because otherwise, system (16) is trivial. Then, let us denote ES by *x*. From the third equation of system (16), we have

(1) $$I=\frac{rx}{\omega S}.$$

Substituting this into the second Equation (15), we obtain

(2) $$L=\alpha +\beta \left(\frac{\frac{rx}{\omega S}}{\zeta +\frac{rx}{\omega S}}\right).$$

Now, let us solve the steady-state equation for the second Equation (16). Please note that the equation for the steady-state solution for protons implies

(3) $$H=\frac{\delta +q+vH_{0}}{v},$$

hence, *H* is independent of our variables. Therefore, the last component of the second equation of system (16) is equal to −constx, where const is a non-negative constant. Hence, we can omit this part in our calculations by incorporating it in the parameter *d*. Then, we have

(4) $$\begin{array}{cc} 0 & =\frac{dx}{dt}\\ \Leftrightarrow 0 & =b\mu S-dx-am\mu px\left(\alpha +\beta \left(\frac{\frac{rx}{\omega S}}{\zeta +\frac{rx}{\omega S}}\right)\right)\\ \Leftrightarrow 0 & =b\mu S\left(\zeta +\frac{rx}{\omega S}\right)-dx\left(\zeta +\frac{rx}{\omega S}\right)-am\mu px\left(\alpha \left(\zeta +\frac{rx}{\omega S}\right)+\beta \frac{rx}{\omega S}\right)\\ \Leftrightarrow 0 & =b\mu S\left(\zeta S+\frac{rx}{\omega }\right)-dx\left(\zeta S+\frac{rx}{\omega }\right)-am\mu px\left(\alpha \left(\zeta S+\frac{rx}{\omega }\right)+\beta \frac{rx}{\omega }\right)\\ \Leftrightarrow 0 & =-x^{2}\frac{r}{\omega }\left(d+am\mu p(\alpha +\beta )\right)+xS\left(b\mu \frac{r}{\omega }-d\zeta -am\mu p\alpha \zeta \right)+b\mu \zeta S^{2} \end{array}$$

By treating the last Equation (4) as a quadratic function of *x*, we can determine the number of candidates for the steady states. In fact, we know that we have two roots, since

(5) $$\Delta =S^{2}{\left(b\mu \frac{r}{\omega }-d\zeta -am\mu p\alpha \zeta \right)}^{2}+4b\mu \zeta S^{2}\frac{r}{\omega }\left(d+am\mu p(\alpha +\beta )\right)>0.$$

From Vieta’s formula, the roots have opposite signs:

(6) $$x_{1}x_{2}=-\frac{b\mu \zeta S^{2}}{\frac{r}{\omega }\left(d+am\mu p(\alpha +\beta )\right)}<0.$$

Finally, the equations for the roots are

(7) $$\begin{array}{cc} x_{1,2} & =\frac{-S\left(b\mu \frac{r}{\omega }-d\zeta -am\mu p\alpha \zeta \right)\pm S\sqrt{{\left(b\mu \frac{r}{\omega }-d\zeta -am\mu p\alpha \zeta \right)}^{2}+4b\mu \zeta \frac{r}{\omega }\left(d+am\mu p(\alpha +\beta )\right)}}{-2\frac{r}{\omega }\left(d+am\mu p(\alpha +\beta )\right)}\\ \; & =c_{1}S, \end{array}$$

where $c_{1}>0$ for the positive root, since we assumed S>0.

Now, let us look at the first equation of system (16). For simplicity purposes, let us transform the third equation of system (16) into

(8) $$I=\frac{rE}{\omega }.$$

Hence, we have

(9) $$L=\alpha +\beta \left(\frac{rE}{\zeta \omega +rE}\right)$$

and we can calculate the second nullcline:

(10) $$\begin{array}{cc} 0 & =\frac{dS}{dt}\\ \Leftrightarrow 0 & =\phi S\left(1-\frac{S}{K}\right)-av_{c}m\mu \left(1-pL\right)x\\ \Leftrightarrow x & =\frac{\phi S\left(1-\frac{S}{K}\right)}{av_{c}m\mu \left(1-p\left(\alpha +\beta \frac{rE}{\zeta \omega +rE}\right)\right)} \end{array}$$

Since we are looking for steady states, we can assume that E≡const, i.e., Equation (10) is equivalent to

(11) $$x=c_{2}S\left(1-\frac{S}{K}\right),$$

where $c_{2}$ is a positive constant. From (7) and (11), we deduce that there are two possible phase portraits, which are dependent on the values of $c_{1}$ and $c_{2}$. In particular, the S-coordinates of the steady states are the solutions of the equation

(12) $$c_{1}S=c_{2}S\left(1-\frac{S}{K}\right).$$

From Equations (12) and (7), (0,0) is one of the steady states. The other one is positive if and only if

(13) $$\begin{array}{cc} S=\left(1-\frac{c_{1}}{c_{2}}\right)K & >0\\ \Leftrightarrow c_{1} & <c_{2}. \end{array}$$

Concluding, our system (16) can have either one or two non-negative steady states. The steady state at the origin always exists. The existence of the positive steady state $\left(S^{*},ES^{*}\right)$ depends on the model’s parameter values in a complex way. □

**Figure 1:**
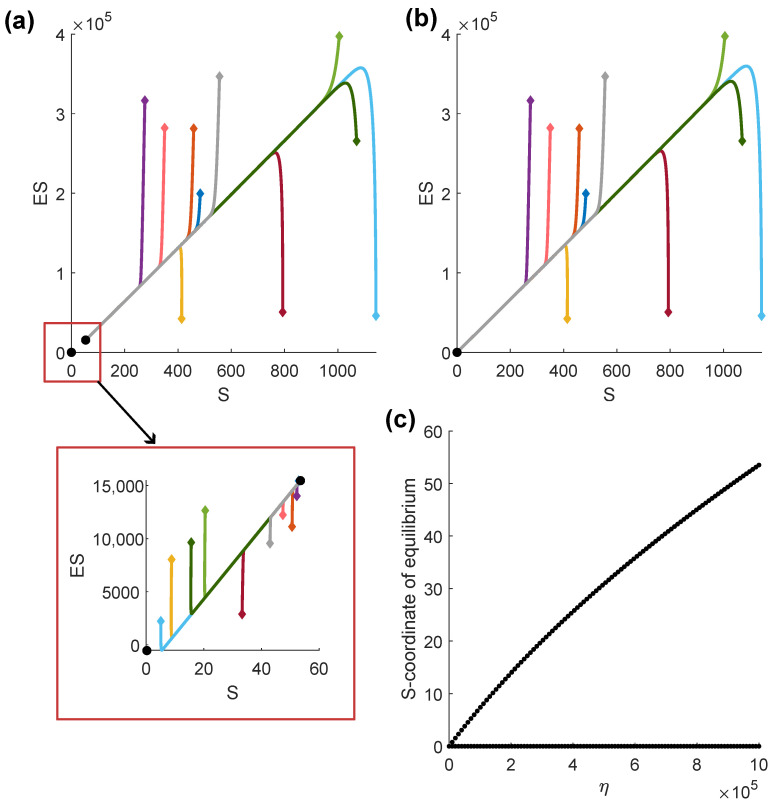
Phase portraits of the simplified model (16). Black dots denote the steady states. The colored lines represent distinct solutions starting from the initial conditions, which are marked with diamond shapes. (**a**) Phase portrait of the CAIX-expressing tumor, including an inset, which shows the behavior of the solutions starting close to the steady states. (**b**) Phase portrait of the CAIX-expressing tumor. (**c**) Influence of parameter eta on the S-coordinate of the steady state.

Having inferred the possible existence of two qualitatively different phase portraits, we wanted to investigate whether they were attainable for plausible biological parameters. For illustration purposes, we set the free parameters to the values obtained from the data fitting procedure (see Table 1), with the exception of the parameters *b* and eta. The first reason for modifying these parameters is that complete tumor eradication, i.e., one of the phase portrait types, was not observed in the data that we used for model calibration. Hence, to obtain this phase portrait, we increased the T-cell infiltration to $b=3\times 10^{4}$. Please note that this may correspond either to a more immunogenic tumor than the one investigated in the in vivo experiments, or an immune response that had been boosted by immunotherapy. Furthermore, we increased the impact of TME acidification on the T-cell population to $\eta =10^{6}$ to widen the distance between the steady states and make the figure more legible. However, the phase portraits were qualitatively the same for smaller values of the parameter eta, as shown in Figure 1c. The zero and the positive equilibrium exist for all simulated values of parameter η. With decreasing eta, the S-coordinate of the steady state decreases toward zero. Due to the large ranges on the phase portrait axes, we opted for a large η-value for the exemplary phase portrait to ensure that the steady states were easily distinguishable on the plot.

We were able to obtain both types of phase portraits by only manipulating the parameter denoting CAIX expression on cancer cells. As shown in Figure 1, we can obtain two non-negative steady states for the parameter value $q=7.6258\times 10^{-13}$, corresponding to the acidification of the TME to ca. 6.6, which falls within the boundary values reported in the literature [18]. The solutions tend to the positive equilibrium. On the other hand, when simulating a CAIX KO tumor, i.e., setting CAIX=0, we obtain one asymptotically stable steady state at the origin, indicating the elimination of the CAIX KO tumor.

### 2.2. Synergistic Combination Therapy with Anti-CAIX and Immune Checkpoint Inhibitors

In the previous section, we showed an example of how CAIX KO tumors might have a drastically different outcome than CAIX-expressing tumors, especially in the presence of a strong immune response. However, in clinical practice, therapy is usually not given indefinitely. Motivated by the promising initial results obtained for the simplified model, we investigated transient combination therapy with anti-CAIX and immune checkpoint inhibitors using the full model (14). We calibrated the model with the parameters resulting from the data-fitting procedure (see Table 1) and standardized the initial conditions by setting inocCells=0.1 to facilitate therapy comparison. Then, we simulated different treatments on this exemplary tumor. First, we let the tumors grow for twenty days prior to initiating any treatment. Then, the treatment with parameters $d_{3}=1$, $d_{1}=0.4$, and $d_{2}=4$ was simulated for a finite duration. Finally, we observed tumor growth until day 200 after inoculation. The control tumor (treatment-free) grew until reaching a maximum volume of 1179 mm${}^{3}$ on day 40.

**Figure 2:**
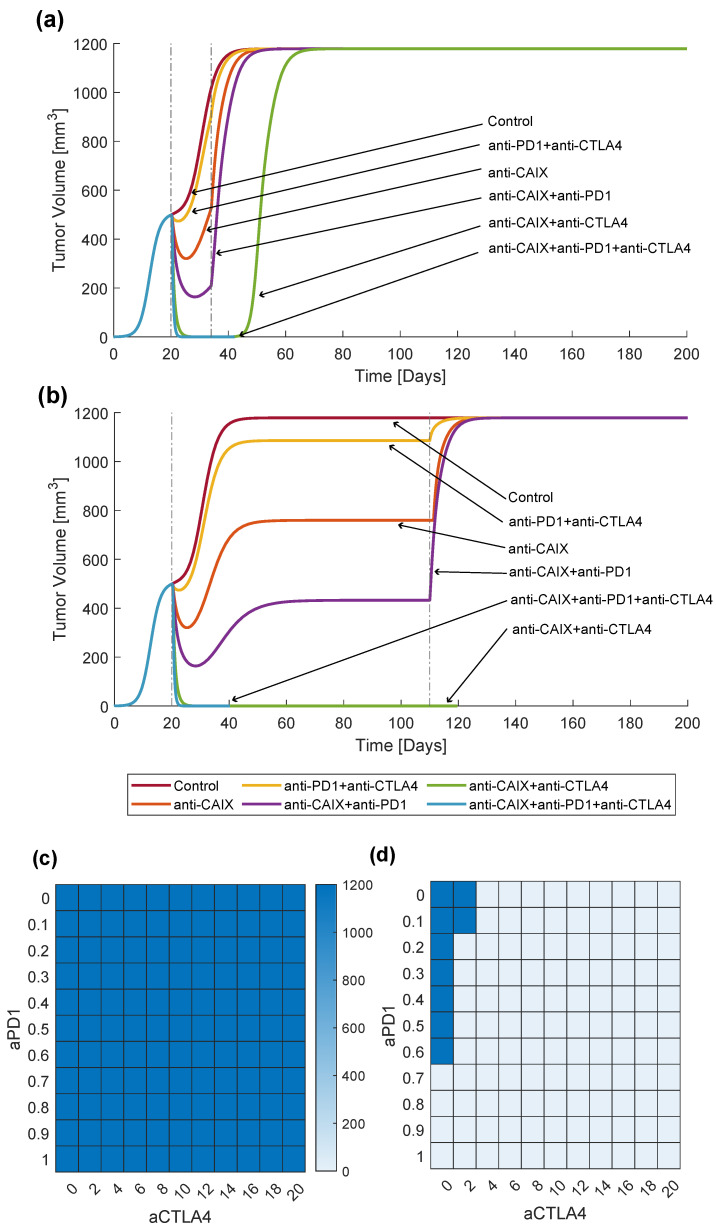
Comparison of the effectiveness of treatment combinations for shorter and longer durations. The lineplots correspond to treatment with the parameters $d_{3}=1$, $d_{1}=0.4$, and $d_{2}=4$. Vertical lines mark treatment start and end. The heatmaps show the tumor volume at day 200 for increasing doses of the combination treatment with anti-PD-1 and anti-CTLA-4. (**a**) Long-term outcome of two weeks of therapy. (**b**) Long-term outcome of 3 months of therapy. (**c**) Volumes of CAIX-expressing tumors for various anti-PD-1 and anti-CTLA-4 doses on day 200 after inoculation. (**d**) Volumes of CAIX KO tumors for various anti-PD-1 and anti-CTLA-4 doses on day 200 after inoculation.

Figure 2a shows the effects of 2-week treatment simulations. Monotherapy with anti-CAIX, combination therapy with two immune checkpoint inhibitors (anti-PD-1 and anti-CTLA-4), as well as combination therapy with anti-CAIX and anti-PD-1 resulted in a short decrease in tumor volume, followed by renewed growth. The tumor growth accelerated upon stopping treatment, and the tumor eventually reached the same size as the control tumor. Combining anti-CAIX and anti-CTLA-4 resulted in tumor recurrence about 2 weeks after treatment suspension, even after seemingly eradicating the tumor during the therapy window. On the other hand, combining anti-CAIX with both immune checkpoint inhibitors decreased tumor size substantially during the treatment time window, and this decrease in size continued after therapy ended, resulting in complete tumor elimination.

To ensure that the scenario presented above was not just a result of short treatment duration, we simulated the same treatments for a longer time period. In Figure 2b, we can see the effect of the same treatments given for 90 days. Here, we can infer that a lack of complete and durable response for treatments that did not combine both anti-CAIX and ICI was not due to the short treatment window. The increased treatment duration induced tumor volume stabilization during treatment. However, tumor growth immediately resumed after stopping the treatment, reaching the size of the control tumor. Hence, prolonging the treatment did not impact the long-term outcome. Conversely, longer treatment with a combination of anti-CAIX and anti-CTLA-4 led to a response that continued after stopping treatment, as opposed to the two-week treatment.

These observations motivated us to hypothesize that in the presence of sufficient CAIX expression that leads to a significant acidification of the TME, combining anti-CAIX with a high enough dose of ICI is necessary for a complete and durable response, regardless of ICI type. To initially test this hypothesis, we analyzed the long-term outcomes of combination therapy with varying doses of anti-PD-1 and anti-CTLA-4 in CAIX-expressing and CAIX KO tumors. Treatment was again given for three months (90 days) and tumor volume was measured on day 200 after inoculation, i.e., another 90 days after the end of therapy. In Figure 2c, we can see that, in fact, no dose of ICI elicited durable response in the CAIX-expressing tumors. All therapy regimens resulted in tumor growth to virtually the same size. On the other hand, as shown in Figure 2d, monotherapy with either ICI or combination therapy with both ICIs resulted in a lack of tumor regrowth after treatment end in CAIX KO tumors, provided that the ICI dose was sufficiently high.

### 2.3. Model Fitting

**Figure 3:**
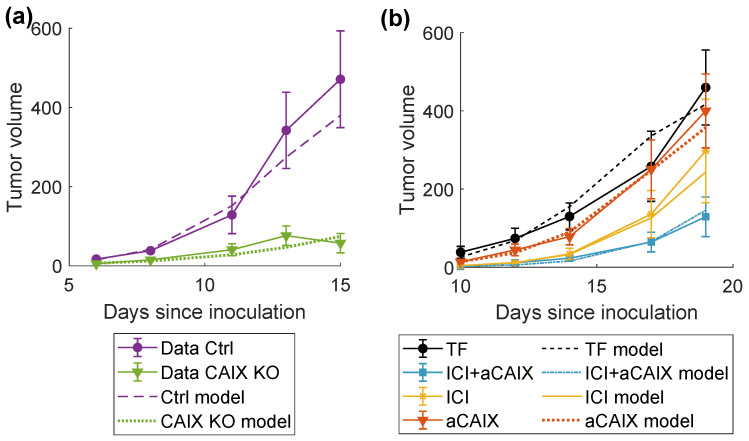
Results of the data-fitting procedure. Markers denote the mean of the experimental data. Error bars represent standard error of the mean. (**a**) Fit of the model to the data on tumor growth for CAIX-expressing (Ctrl) and CAIX KO cells. (**b**) Fit of the model to the data on the comparison of treatment combinations. TF: treatment free; ICI: anti-PD-1+anti-CTLA-4; aCAIX: anti-CAIX.

**Figure 4:**
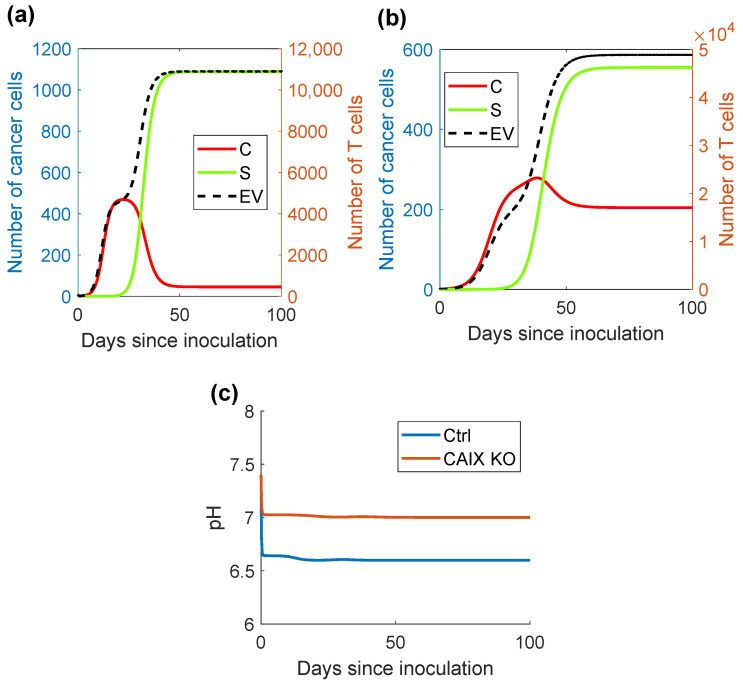
Comparison of a CAIX-expressing and a CAIX KO tumor: (**a**) Number of cells in the model with CAIX expression. (**b**) Number of cells in the CAIX KO model. (**c**) Comparison of the pH level between the CAIX-expressing (Ctrl) and the CAIX KO models.

Finally, we wanted to investigate whether our model could correctly replicate experimental findings. We were able to obtain a good fit for the in vivo data, as shown in Figure 3a,b. The final values of the free parameters are shown in Table 1. The loss function was 1.32. Figure 3a confirms the conclusions in [16] that knocking out CAIX expression significantly decreases tumor growth. However, the in vivo experiments showed only the beginning of the tumor growth and did not show any sign of stabilization of the tumor volume. To gain a deeper understanding of the system’s behavior, we performed simulations with the calibrated model for longer time spans. Figure 4c shows that our model confirmed that CAIX acidifies the TME, lowering the pH steady state from approximately 7 to 6.6. Furthermore, Figure 4a,b indicate that the tumor volume and the number of infiltrating T-cells stabilized after around 60 days at ca. 759 mm${}^{3}$ for the CAIX KO tumor and 1179 mm${}^{3}$ for the CAIX-expressing tumor. Strikingly, there was a significant difference in the ratio of CSC to CC between both simulations, with the CAIX KO tumor having a decreased CSC fraction.

## 3. Discussion

Herein, we have introduced a new mathematical model of tumor–immune interactions and applied it to investigate the effectiveness of immunotherapy and CAIX inhibition. Our model suggests a synergistic combination of anti-CAIX with immune checkpoint inhibitors. Importantly, we have shown that immunotherapy alone or anti-CAIX monotherapy might lead to tumor recurrence after treatment interruption. On the other hand, combining immune checkpoint inhibitors with anti-CAIX might change the TME in favor of the immune cells and elicit a complete and durable response. However, this required a sufficiently boosted immune response. Combining low doses of immunotherapy with CAIX suppression resulted in tumor regrowth after stopping the therapy. Interestingly, for low ICI doses combined with CAIX inhibition, we observed non-obvious simulation results, such as pseudo-elimination, followed by disease recurrence long after ending treatment.

Our main finding was that adding short-term CAIX inhibition can turn partial and temporary responses to immune checkpoint inhibitors into a response that continues after treatment until tumor eradication. This is a significant observation since immune checkpoint inhibitors have relatively low response rates, and improving them would fundamentally change the outlook for countless cancer patients. Importantly, we observed this synergistic combination only for tumors with efficient immune responses. Hence, choosing the optimal ICI dosage remains vitally important. A limitation worth noting is that our model is not defined in the absence of a tumor. Due to numerical precision, some of our simulations in Figure 2 reached a tumor volume of zero, forcing a premature end of the simulation to avoid division by zero. Therefore, we cannot predict what happens afterward, so we cannot state for sure how durable the response would be. Regardless, we can see that in some cases of combination treatment with anti-CAIX and a high enough dose of ICIs, the treatment response continues after treatment, as opposed to monotherapies, showing a synergistic instead of just an additive effect. These initial simulations imply that a combination of ICIs and anti-CAIX might lead to a more durable response in CAIX-expressing patients and allow for a more relaxed treatment schedule.

Moreover, we have proven analytically that a simplified yet realistic model version may have either one or two steady states, depending on the parameters. One of the equilibria denotes tumor eradication, while the other corresponds to stable disease. Numerically, we have shown that CAIX expression has a pivotal impact on the stability of the tumor-free equilibrium. Provided an abundant immune response, which might be achieved via immunotherapy, CAIX KO tumors tend to the zero equilibrium, whereas their CAIX-expressing counterparts stabilize at the positive steady state. Importantly, the simplified version of the model overcomes the problem of instability close to zero values of tumor volume.

Another interesting observation was that the data-fitting procedure revealed a lower CSC fraction in the CAIX KO tumor. This was probably due to the subdued immune response in the acidic TME, which diminished T-cell-mediated cancer cell killing. Therefore, the apoptotic death of CCs had a graver impact on the number of CCs in the acidic tumor than T-cell-mediated killing. This observation was based on calibrating the model via a small data set, so we cannot draw any definite conclusions. Nevertheless, it shines a light on an interesting research topic. CSCs are believed to be the main drivers of tumor progression and treatment recurrence, and yet they seem to be resistant to most conventional treatments. In lung cancer, for example, chemotherapy failure is commonly believed to be due to CSC resistance [19]. Interestingly, some pre-clinical studies show that CAIX inhibition sensitizes previously resistant tumor cells to chemotherapy. In [20], Lock et al. observed reduced lung cancer growth and metastasis, when combining paclitaxel with anti-CAIX. If the effectiveness of CAIX inhibition on CSC elimination were confirmed, it would be a promising combination therapy target with chemotherapy. Lock et al. have reported CSC depletion due to CAIX inhibition in orthotopic breast tumor models, but also a lack of CSC expansion in vitro when coupled with CAIX inhibition, indicating that immune-unrelated mechanisms are at play as well [20]. However, the impact of the different mechanisms of CSC depletion via CAIX is not yet fully elucidated. For future research, our model could be expanded to allow for further investigation of the intricate relationships between CSCs and CAIX.

Finally, our model can reproduce experimental in vivo data with and without treatment. It should be noted that our model is now only calibrated with a small data set, produced by taking the average results instead of the individual repeats from the in vivo experiments. This was sufficient for our exploratory analysis that aimed at establishing hypotheses concerning the combination of CAIX inhibition and ICI therapy and initially validating the model. More data concerning tumor growth in CAIX expressing and CAIX KO tumors are needed to further validate the model. In particular, currently we do not know whether our model generalizes well to unseen data. If more data were available, we could test the prediction capabilities of the model and refine it, e.g., by using regularization techniques to minimize the danger of overfitting (see, for example, ref. [21] for an overview of techniques that can be used to select the most vital parameters for the data-fitting procedure). We see two possibilities for testing the model’s prediction capabilities. For example, longer data sets could be used to calibrate the model on initial time points and then test its prediction accuracy on unseen data points. Alternatively, if data from the individual repeats were available, it would be possible to calibrate the model using a subset of the data sets, and then validate it using the remaining ones. Due to this lack of data, we have opted to model the treatments by assuming a constant inhibition effect for the entire treatment duration. In reality, drugs are removed from the body and their effect tapers off over time. If our model were to be used to compare different treatment schedules and identify optimal treatment protocols, the pharmacokinetics of the drugs should be included in the equations. However, this would increase the number of model parameters and thus require more data for calibration.

## 4. Materials and Methods

### 4.1. Differential Equation Model Formulation

Our model’s domain is the tumor itself. Therefore, the variables describing cancer cells denote volumes, whereas other variables describe the density or concentration of the described cell type or substance in the tumor. Let *C* and *S* denote the volume of cancer cells and cancer stem cells (${\mathrm{mm}}^{3}$), respectively. Let *E* denote the density of active T-cells in the tumor ($\frac{\mathrm{cells}}{{\mathrm{mm}}^{3}}$). Let *I* and *H* denote the IFNγ and proton concentrations in the tumor ($\frac{\mathrm{pg}}{{\mathrm{mm}}^{3}}$, $\frac{\mathrm{mol}}{{\mathrm{mm}}^{3}}$), respectively. Let the fraction of PD-L1-expressing cancer cells be denoted by *L*. Finally, assuming that T-cells do not significantly contribute to the tumor volume, let

V(t)=C(t)+S(t)

be the tumor volume. This assumption is a certain simplification based on the fact that the diameter of T-cells is much smaller than that of cancer cells, and that cancer cells outnumber lymphocytes in the tumor. Then, instead of modeling the temporal changes in the densities *E*, *I*, and *H*, we can model the temporal changes in the number of cells and molecules by multiplying the densities by the tumor volume, obtaining equations with respect to the products E·V, I·V, and H·V. Hence, we consider the following system of differential equations:

(14) $$\left\{\begin{array}{c} \begin{array}{cc} \frac{dC}{dt} & =f\left(C\right)+\sigma f\left(S\right)-av_{c}m\mu \left(1-p(1-d_{1})L\right)C\cdot E-nC\\ \frac{dS}{dt} & =\left(1-\sigma \right)f\left(S\right)-av_{c}m\mu \left(1-p(1-d_{1})L\right)S\cdot E\\ \frac{dE\cdot V}{dt} & =b\mu \left(1+d_{2}\right)V-dE\cdot V-am\mu p\left(1-d_{1}\right)LV\cdot E\\ \; & -\eta E\cdot V\max\left(1-\frac{H_{thresh}}{H},0\right)\\ \frac{dI\cdot V}{dt} & =rE\cdot V-wI\cdot V\\ \frac{dH\cdot V}{dt} & =\delta \left(C+S\right)+q\left(1-d_{3}\right)\left(C+S\right)-v\left(H-H_{0}\right)\cdot V, \end{array} \end{array}\right.$$

where

(15) $$\begin{array}{cc} f(X) & =\phi X\cdot \left(1-\frac{V}{K}\right),\\ \mathrm{and}\;L & =\alpha +\beta \frac{I}{\zeta +I},\beta <=1-\alpha \end{array}$$

for X=C or X=S. The parameters pertaining to treatment effectiveness ($d_{i}$ for i=1,2,3) or CAIX expression (*q*) are assumed to be non-negative. All other parameters are positive. Moreover, we assert V>0, because the system assumes tumor existence.

The model is based on the following assumptions:

1. *Tumor growth* Tumor growth is logistic with the carrying capacity *K*. The model differentiates between cancer non-stem cells (denoted in the manuscript as CCs) and cancer stem cells ( denoted as CSCs). CSCs can only be killed by immune cells, whereas CCs experience apoptosis with the rate *n* [22]. CSCs divide asymmetrically with rate σ and symmetrically otherwise.
2. *Tumor–immune interactions* T-cells’ infiltration is proportional to the tumor volume, and their number EV decreases exponentially due to cell death. They attack and kill cancer cells at a rate proportional to their density in the tumor, as proposed in [23]. Notably, this is a spin on the classical Kuznetsov-type interactions as presented in [24], where tumor cell killing is proportional to the product of the number of tumor cells and T-cells. We believe that our modification suits our needs better than the original interaction term. In particular, let us consider the scenario in which we compare two tumors consisting of the same number of CCs and T-cells. Let us assume further that one of the tumors also has a large population of CSCs, while the second has none. If we used the original Kuznetsov-type term, the CC decay due to interactions with T-cells would be the same in both tumors. On the other hand, in our model, the decay of CCs is smaller in the tumor with CSCs, since the T-cell density is smaller in this tumor. This seems more plausible, as the lymphocytes are then more likely to attack CSCs instead of just CCs. However, our model tacitly assumes that the tumor infiltration by lymphocytes is not over-saturated, i.e., the interactions between T-cells and cancer cells are not limited by a lack of cancer cells. In particular, T-cell decay due to interactions with cancer cells depends only on the number of T-cells. Finally, only cancer cells expressing MHC class I on their surface are recognized and attacked by T-cells. Moreover, the immune response is higher for tumors with a higher tumor mutational burden. Therefore, the rate of tumor cell killing is equal to amμ, where *a* indicates the interaction rate between tumor and immune cells, *m* the fraction of cancer cells expressing MHC class I, and μ quantifies TMB, as proposed in [25].
3. *PD-1-PD-L1 pathway* Tumor cell killing by immune cells is inhibited via the binding of PD-1 and PD-L1, which induces T-cell anergy. We assume that the fraction of PD-1-expressing cells is constant and equal to *p*. The expression of PD-L1, however, can be either constitutive or adaptive, i.e., induced by IFNγ as a way of escaping the immune response [26]. We assume that the fraction of cancer cells with constitutive PD-L1 expression is constant and equal to α. Adaptive PD-L1 expression is dynamic and bounded from above by the parameter β.
4. *Substances in the TME* IFN-γ is produced by active lymphocytes with rate *r* and decays naturally with rate ω. Protons are produced due to cancer cell metabolism with rate δ and due to CAIX expression with rate *q*. Outside of the tumor, we assume a physiological pH. The flux of protons into and out of the TME is proportional to the difference between the pH in the TME and the physiological pH. Immune cells that are exposed to acidosis die. The lower the pH, the greater the induced death rate.

Additionally, the model considers treatment with three inhibitors: anti-CAIX, anti-PD-1, and anti-CTLA-4. The treatment is modeled with the following assumptions:

1. Anti-CAIX suppresses CAIX expression by the fraction $d_{3}$.
2. Anti-PD-1 suppresses PD-1 expression by the fraction $d_{1}$.
3. Anti-CTLA-4 is mainly responsible for reinvigorating early T-cell activation in the lymph nodes, which we include in our model by increasing lymphocyte influx by the rate $d_{2}$.

For qualitative analysis, we consider a simplified version of the model, which is two-dimensional and allows for a phase portrait analysis. Here, we assume that all cancer cells are stem cells. The reason for choosing CSCs instead of CCs is that those are the cells that are crucial to treatment success or failure. Moreover, since the production and decay of protons and IFN-γ are much faster than cell actions, we may assume that protons and IFN-γ are in their steady states. For simplicity purposes, we will analyze the treatment-free version of the model, but please note that the phase portrait analysis also works for the model that includes treatment. In this case, the effect of treatment can be included in the parameters *b*, *p*, and δ. The treatment-free simplified version of the model looks as follows:

(16) $$\left\{\begin{array}{c} \begin{array}{cc} \frac{dS}{dt} & =f\left(S\right)-av_{c}m\mu \left(1-pL\right)S\cdot E,\\ \frac{dE\cdot S}{dt} & =b\mu S-dE\cdot S-am\mu pLE\cdot S-\eta E\cdot S\max\left(1-\frac{H_{thresh}}{H},0\right)\\ 0 & =rE\cdot S-wI\cdot S\\ 0 & =\delta S+qS-v\left(H-H_{0}\right)\cdot S. \end{array} \end{array}\right.$$

### 4.2. Model Calibration

Most of the model’s parameters were calibrated with values found in the literature, see Table 2. However, a few parameters could not be determined from the literature and were denoted as free parameters. To estimate their value, we fitted our model to experimental data presented in [16]. In particular, we took average data representing the growth of B16F10 cell lines with and without CAIX expression, as well as the growth of four treatment cohorts: cells treated with the CAIX inhibitor SLC-0111 (aCAIX), anti-PD-1 and anti-CTLA-4 (ICI), combination therapy of anti-CAIX, anti-PD-1, and anti-CTLA-4 (aCAIX+ICI) and a treatment-free control group (TF). Tumor volume was measured on days 6, 8, 11, 13, and 15 when comparing CAIX-expressing and CAIX KO cell lines, and on days 10, 12, 14, 17, and 19 for the comparison of different treatments. This yielded six data sets of five data points each. In the in vivo experiments, $5\times 10^{5}$ tumor cells in 100 μL PBS were inoculated subcutaneously onto the back of female C57Bl/6J mice. In our in silico simulations, we assume that only the fraction inocCells of these $5\times 10^{5}$ tumor cells initiates the tumor and that a fraction *CSCrat* of these initiating tumor cells are CSCs. Model calibration was performed based on all data sets simultaneously. All parameters except inocCells were asserted to be the same for each data set. We allowed the fraction inocCells to be different for each data cohort to account for the variability of inoculation effectiveness and initial conditions in distinct experiments. In our model, CAIX suppression was modeled by setting the parameter CAIX to zero. Similarly, lack of treatment was modeled by setting the appropriate treatment parameter to zero ($d_{1}$, $d_{2}$, $d_{3}$). Fitting our model to data was performed using the MATLAB function *lsqnonlin*, i.e., using the least squares method. This function performs simultaneous fitting of all parameters using a subspace trust region method. As the loss function, we took the sum of squares of the residuals, scaled by the data values, i.e., $loss=\sum_{i=1}^{n}{\left(\frac{y_{i}-\hat{y_{i}}}{y_{i}}\right)}^{2}$, where $y_{i}$ denotes the actual data value, $\hat{y_{i}}$ denotes the volume calculated from the model, and n=30 is the number of data points. Data fitting was performed 1000 times by sampling the initial values of the parameters from a multivariate uniform distribution bounded by the upper and lower bounds of the parameter space. The fit with the lowest loss function was selected.

## 5. Conclusions

Here, we have presented a new differential equation model of the impact of acidity and CAIX expression on tumor–immune interactions. Initial calibration with pre-clinical data showed that the model can accurately replicate experimental findings. Analytical and numerical analysis implicates that a combination of CAIX suppression with boosted immune response, e.g., via immune checkpoint inhibitors, is synergistic. Importantly, it can turn a partial response to monotherapies that leads to immediate recurrence after treatment end into a complete response that continues after treatment. In the future, our model could be calibrated with more data to further validate it, investigate the impact of CAIX expression on cancer stem cell dynamics, and compare treatment protocols. In particular, as of now, the model has only been calibrated with murine data, which does not always translate to human studies, so further investigation of these findings is needed.

## Figures and Tables

**Table 1:** Model parameter values resulting from the data-fitting procedure. Asterisks denote substituted variables.

Parameter Name	Value	Lower Bound	Upper Bound
$a^{*}=am\mu$	834.16	0	$10^{3}$
$b^{*}=b\mu$	$5.821\times 10^{3}$	0	$6\times 10^{3}$
eta	$2.485\times 10^{3}$	0	$10^{6}$
n	0.799	0	10
*q*	$7.6258\times 10^{-13}$	$5\times 10^{-13}$	$3\times 10^{-12}$
$d_{1}$	0.028	0	1
$d_{2}$	0.011	0	20
$d_{3}$	0.066	0	1
initCSCrat	$3.083\times 10^{-5}$	0	1
$ic_{ctrl}$	0.210	0	1
$ic_{caixko}$	0.336	0	1
$ic_{v}$	0.044	0	1
$ic_{s}$	0.027	0	1
$ic_{pc}$	0.007	0	1
$ic_{pcs}$	0.004	0	1

**Table 2:** Model parameters. An empty value column denotes free parameters.

Par	Interpretation	Value	Unit	Source
ϕ	maximal rate of tumor cell growth	$\frac{24}{17.2}$	day${}^{-1}$	[27]
**K**	carrying capacity for tumor cells	1200	mm${}^{3}$	permitted tumor volume limit [16]
σ	probability of asymmetric division	0.42	-	[17]
a	interaction rate between tumor cells and TILs	-	day${}^{-1}$	free parameter
$v_{c}$	volume of one tumor cell	$6.2\times 10{}^{-6}$	$\frac{{\mathrm{mm}}^{3}}{\mathrm{cell}}$	[25]
m	mean MHC class I expression	2.3%	-	[25]
μ	antigenicity strength (single nucleotide variations)	908	-	[25]
p	mean PD-1 expression by TILs	54%	-	[25]
n	tumor cell apoptosis rate	-	day${}^{-1}$	free parameter
b	infiltration rate of T-cells into TME	-	$\frac{\mathrm{cells}}{{\mathrm{mm}}^{3}*\mathrm{day}}$	free parameter
d	apoptosis rate of T-cells	0.406	day${}^{-1}$	[25]
η	rate of T-cell death due to acidosis	-	day${}^{-1}$	free parameter
*r*	rate of IFNγ production	$24.48\times 10^{-4}$	$\frac{\mathrm{pg}}{\mathrm{cell}*\mathrm{day}}$	[17]
ω	rate of IFNγ decay	2.4	day${}^{-1}$	[17]
δ	rate of proton production due to tumor cell metabolism	$3\times 10^{-13}$	$\frac{\mathrm{mol}}{{\mathrm{mm}}^{3}*\mathrm{day}}$	assumption to yield realistic pH values
q	rate of proton production due to CAIX expression	-	$\frac{\mathrm{mol}}{{\mathrm{mm}}^{3}*\mathrm{day}}$	free parameter
v	rate of proton flux into and out of the TME	5	day${}^{-1}$	assumption to yield realistic pH values
$H_{0}$	proton concentration at physiological pH	3.98 × 1 ×$10^{-14}$	$\frac{\mathrm{mol}}{{\mathrm{mm}}^{3}}$	
$H_{thresh}$	proton concentration equivalent to pH = 6.7	2 × 1 ×$10^{-13}$	$\frac{\mathrm{mol}}{{\mathrm{mm}}^{3}}$	[17]
α	constitutive PD-L1 expression	0.1	-	[17]
β	rate of adaptive PD-L1 expression	0.1	-	assumption, <1−α
ζ	saturation constant	$0.01\times 10^{-3}$	$\frac{\mathrm{pg}}{{\mathrm{mm}}^{3}}$	assumption
$d_{1}$	effect of anti-PD-1	-	-	free parameter
$d_{2}$	effect of anti-CTLA-4	-	-	free parameter
$d_{3}$	effect of anti-CAIX	-	-	free parameter
CSCrat	ratio of CSC at inoculation	-	-	free parameter
inocCells	ratio of inoculated cells that initiates the tumor	-	-	free parameter

## Data Availability

Data sharing not applicable.

## Abbreviations

The following abbreviations are used in this manuscript:

CAIX	carbonic anhydrase IX
PD-1	programmed cell death protein 1
CTLA-4	cytotoxic T-lymphocyte-associated protein 4
CC	cancer cells
CSC	cancer stem cells
TME	tumor microenvironment
KO	knock-out
NSCLC	non-small-cell lung cancer
